# Comprehensive analysis of the functional and immunological significance of ETV4 in pan-cancer and its validation in digestive tumors

**DOI:** 10.3389/fimmu.2025.1595850

**Published:** 2025-05-21

**Authors:** Lingli Huang, Xujia Li, Silan Huang, Qi Jiang, Chang Jiang, Wenzhuo He, Yuchen Cai, Guifang Guo

**Affiliations:** ^1^ VIP Department, Sun Yat-sen University Cancer Center, Guangzhou, China; ^2^ State Key Laboratory of Oncology in South China, Sun Yat-sen University Cancer Center, Guangzhou, China; ^3^ Collaborative Innovation Center for Cancer Medicine, Sun Yat-sen University Cancer Center, Guangzhou, China; ^4^ Guangdong Provincial Clinical Research Center for Cancer, Sun Yat-sen University Cancer Center, Guangzhou, China; ^5^ Experimental Research Department, State Key Laboratory of Oncology in South China, Sun Yat-sen University Cancer Center, Guangzhou, China

**Keywords:** Etv4, pan-cancer, immunotherapy, immune-related molecules, FGL-1

## Abstract

**Background:**

The E26 transformation-specific (ETS) transcription factor family is widely expressed and implicated in tumorigenesis. Among them, ETV4 plays a crucial role in cancer progression. However, its broader impact on prognosis and immune regulation across different malignancies remains insufficiently understood.

**Methods:**

Based on public databases and our experimental validation, we systematically investigated the role of ETV4 in various cancers. Cytoscape, GSCALite, CancerSEA, STRING, HPA, TIGER, TISIDB, and R were used to assess ETV4’s expression and functional impact on basis of the TCGA and GTEx databases. Experimental validation included a range of methods such as CCK8 assays, clonogenic assays, migration assays, flow cytometry, RT-qPCR, immunohistochemistry (IHC), lentiviral transfection, and *in vivo* tumor formation assays.

**Results:**

ETV4 overexpression was detected in several cancer types and was associated with poor prognosis and specific molecular and immune subtypes. ETV4 was linked to overall survival in 10 of them. Furthermore, ETV4 played a key role in modulating multiple signaling pathways and was associated with immune regulation, particularly in melanoma and renal cell carcinoma, where its expression predicted immune responses. Knockdown of ETV4 in digestive tumors inhibited cell proliferation and migration, promoted apoptosis, and altered the expression of immune-related molecules. Further *in vitro* and *in vivo* analyses revealed that knockdown of ETV4 led to significant downregulation of FGL1 expression in BxPC3 cells and in tumors from Panc02 xenograft models. Kaplan-Meier Plotter analysis showed that lower FGL1 expression was associated with longer overall survival in patients receiving anti-PD1 therapy. In silico analysis using NCBI and UCSC genome databases further identified ETV4 as a putative transcription factor that may bind to the FGL1 promoter region, suggesting a potential regulatory relationship.

**Conclusions:**

ETV4 shows differential expression across various cancer types and may serve as a potential prognostic biomarker in certain tumor types. Further validation in clinical samples and functional studies is warranted to clarify the biological role of ETV4 and its potential utility as a therapeutic target or prognostic indicator in pan-cancer.

## 1 Introduction

Cancer remains a leading cause of mortality and morbidity worldwide, imposing a significant economic burden on society (1). Current treatment options—including chemotherapy, radiotherapy, targeted therapies, immunotherapies, and surgical interventions—have advanced considerably in recent years (2). However, challenges such as drug resistance, metastasis, and immune evasion continue to limit the effectiveness of these treatments (3). Consequently, there is an urgent need to identify novel biomarkers and therapeutic targets to improve cancer outcomes.

Tumorigenesis, progression, and response to therapy are intricately linked to interactions with the host immune system (4). The effectiveness of the immune response is shaped by numerous host and environmental factors that influence the intensity and duration of anti-cancer activities, many of which are dependent on intact mitochondrial metabolism (4, 5). Mitochondria play a central role in tumorigenesis, not only by providing bioenergetic support but also by regulating anabolic and catabolic processes, transcription, and cell death (6). The E26 transformation-specific (ETS) transcription factor family has been shown to modulate oxidative stress, impacting mitochondrial function and, consequently, the efficacy of cancer treatments (7). Furthermore, the ETS family is critical for cellular growth, differentiation, and transformation, and plays a key role in tumor invasion and metastasis (8).

ETV4 (also known as E1AF and PEA3), along with ETV1 (ER81) and ETV5 (ERM), constitutes the PEA3 subfamily of ETS transcription factors. ETV4 is characterized by its ETS DNA-binding domain, which recognizes the GGAA/T core consensus motif (9, 10). ETV4 is involved in various biological processes, including embryogenesis and the development of the hippocampus and kidneys, through the activation of target gene transcription (11). In cancer, ETV4 regulates genes that control cell proliferation and migration and can also activate multiple matrix metalloproteinase (MMP) genes to promote cancer cell invasion (9, 12). ETV4 promotes cell proliferation and invasion through inducing the activity of LAIR1 promoter (13). In most cases, ETV4 is implicated in carcinogenesis and participates in the process of tumor metastasis (14–17). ETV4 has been implicated in carcinogenesis and tumor metastasis, with its deregulated target genes playing a critical role in these processes. However, previous studies on ETV4’s function have been limited to specific cancer types. Recent research has also revealed that ETV4 plays a crucial role in modulating the tumor microenvironment, particularly by upregulating PD-L1 and CCL2, which leads to increased infiltration of immunosuppressive cells such as tumor-associated macrophages (TAMs) and myeloid-derived suppressor cells (MDSCs). This immunosuppressive environment not only facilitates tumor metastasis but also presents significant challenges to the effectiveness of immunotherapies (18). As a result, ETV4 could potentially serve as a biomarker for predicting immunotherapy responses and as a target for enhancing the efficacy of immune checkpoint inhibitors. Thus, a comprehensive analysis of ETV4 across a pan-cancer dataset is essential to uncover new strategies for cancer treatment.

In this study, we explored the expression and prognostic value of ETV4 across various cancers. Using multiple public databases, we investigated the role of ETV4 in the immune response and examined potential associations between ETV4 expression and immune-related molecules in different cancer types. Additionally, we analyzed the biological functions and pathways associated with ETV4 through KEGG and GSEA analyses. Our findings revealed that ETV4 overexpression is significantly associated with poor prognosis in a range of cancers and regulates cancer progression through multiple mechanisms. Furthermore, we validated these findings in digestive tumors, demonstrating that ETV4 expression was closely related to tumor cell proliferation, migration, and apoptosis, and significantly influences the expression of immune-related molecules, highlighting its potential impact on immunotherapy treatments. Moreover, we further validated the existence of a correlation between ETV4 and Fibrinogen-like protein 1 (FGL1), a major ligand for the inhibitory receptor LAG3, in pancreatic cancer, suggesting that the ETV4-FGL1 axis may be a key player in tumor immune evasion (19). This study provides valuable insights into the role of ETV4 in cancer and identifies potential strategies to enhance cancer immunotherapy.

## 2 Methods

### 2.1 Public database data analysis

#### 2.1.1 ETV4 expression and datasets acquisition

We analyzed ETV4 expression dysregulation across 33 cancer types by integrating the GTEx (https://gtexportal.org/) and TCGA (https://cancergenome.nih.gov) databases. Samples with an expression value of “0” were excluded to ensure data quality, and paired samples were specifically analyzed to assess differential expression. All data were normalized using log2 transformation for consistency.

#### 2.1.2 Survival analysis of ETV4 across 33 cancer types

Survival outcomes were analyzed using the Kaplan-Meier method with the R “Survival” package. We compared overall survival (OS) between low and high ETV4 expression groups across the cancer spectrum. Statistical significance was assessed using log-rank tests.

#### 2.1.3 Expression of ETV4 in various immune subtypes of cancer

The association between ETV4 expression and cancer immune subtypes was examined using the TISIDB database. This analysis focused on ETV4 mRNA levels across six immune signatures, including IFN-γ dominant, wound healing, lymphocyte depleted, inflammatory, TGF-β dominant, and immunologically quiet types.

#### 2.1.4 Genetic alteration analysis of ETV4

ETV4 genetic alterations were retrieved from the cBioPortal (https://www.cbioportal.org/), including all studies from the TCGA Pan-Cancer Atlas. Mutation details were visualized using the R “maftools” package.

#### 2.1.5 Protein-protein interaction network analysis of ETV4

We utilized the STRING database (https://string-db.org/) to gather potential ETV4 interacting proteins, with further analyses conducted using Cytoscape. The Pathlinker plugin reconstructed the top 10 signaling pathways and mapped pathways from receptors to transcription factors within the PPI network, highlighting significant gene correlations across cancers.

#### 2.1.6 Functional enrichment analysis of ETV4

Functional implications of ETV4 were explored through Gene Ontology (GO) and the Kyoto Encyclopedia of Genes and Genomes (KEGG) pathway analyses using the “clusterProfiler” and “org.Hs.eg.db” R packages. The significance threshold for p-values was set at less than 0.01.

#### 2.1.7 GSCALite analysis

The GSCALite platform was used to integrate pharmacogenomic data and assess significant pathways related to ETV4 activation or inhibition across cancer types, using data from GDSC, CTRP, GTEx, and TCGA.

#### 2.1.8 Gene set enrichment analysis

GSEA was performed to discern biological pathway variations between low and high ETV4 expression groups using “clusterProfiler”. Enrichment results were presented as mountain maps for clear visualization.

#### 2.1.9 Immunogenomic analysis of ETV4 in 33 types of cancers

The ssGSEA algorithm within the “GSVA” package assessed correlations between ETV4 expression and immune-related molecules, with results visualized using the “ggplot2” package in the form of heatmaps.

#### 2.1.10 TIGER database

We utilized the TIGER database (http://tiger.canceromics.org/#/) to predict immunotherapy responses based on ETV4 expression across various cancers (20), integrating extensive gene expression data related to tumor immunology.

### 2.2 Experimental validation

#### 2.2.1 Cell culture and transfection

Human pancreatic cancer cell line (PANC-1, BxPC3), human cholangiocarcinoma cell line (RBE), 293T cells were kept by the laboratory, human liver cancer cell line (SK-Hep1, Hep3B), human breast cancer cell line (MDA-MB-231), human lung cancer cell line (A549, HCC827), the mouse pancreatic cancer cell line (Panc02) were obtained from the cell bank of Sun Yat-sen University Cancer Center. All cell lines were originally purchased from the Guangzhou Cellcook Biotech Co., Ltd. MDA-MB-231 and PANC-1 cells were cultured in DMEM medium, while other cells were maintained in RPMI-1640 medium (Gibco) supplemented with 10% fetal bovine serum (FBS, Gibco, South America) and 1% antibiotics (penicillin and streptomycin, Gibco, USA) at 37°C in a 5% CO_2_ atmosphere.

The shRNA lentiviral ETV4 (sh-ETV4) and negative control (sh-NC) were synthesized by DHbio Technology (Guangzhou, China) (**Supplementary Table S1**). RBE, SK-Hep1, BxPC3, and Panc02 cell lines were used as lentiviral constructs to stably knockdown ETV4. 293T cells were used for lentiviral packaging, and virus-infected target cells were collected and 2ug/mL puromycin screening of target cells for 2 weeks.

#### 2.2.2 Western blot

Total protein was extracted using RIPA buffer (CWBIO, China) supplemented with phosphatase inhibitors. Protein concentrations were measured and adjusted using a BCA Protein Assay Kit (ThermoFisher, China). Proteins were then separated by SDS-PAGE and transferred to PVDF membranes. Membranes were blocked with 5% non-fat milk at room temperature for 1 hour and incubated overnight at 4°C with primary antibodies against GAPDH (Proteintech; Cat #10494-1-AP, China) and ETV4 (Proteintech; Cat #10684-1-AP, China). Horseradish peroxidase (HRP)-conjugated secondary antibodies were applied, and bands were visualized using FDbio-Dura ECL reagent (FDbio, Cat# FD8020, China).

#### 2.2.3 Cell proliferation and clonogenic assay

For proliferation assays, 1000 cells per well were seeded in 96-well plates. After incubation periods of 0, 24, 48, 72, 96, and 120 hours, CCK-8 solution was added according to the manufacturer’s instructions. Absorbance at 450 nm was measured after an additional 2-hour incubation. For the clonogenic assay, cells were plated at a density of 1000 cells per well in six-well plates and cultured for 10–14 days. Colonies were fixed with 4% paraformaldehyde (PFA) and stained with 0.1% crystal violet for 30 minutes.

#### 2.2.4 Cell migration assays

Migration assays were conducted using Transwell chambers (Corning, Cat# 3422, USA) with an 8 µm pore size. Cells (5 x 10^4^) were seeded in the upper chamber, and the lower chamber contained RPMI-1640 supplemented with 10% FBS. After 24 hours at 37°C, non-migrated cells were gently removed, and migrated cells were fixed with 4% PFA and then stained with 0.1% crystal violet, imaged and quantified with ImageJ software.

#### 2.2.5 Apoptosis assay

Cells were collected using EDTA-free trypsin, washed with pre-cooled PBS, and resuspended in 500 μl of 1X binding buffer. Cells were then incubated with 5 µL of annexin V-647 in the dark for 10 minutes followed by 5 µL of propidium iodide (PI) for 5 minutes. Apoptosis was analyzed within 30 minutes by flow cytometry.

#### 2.2.6 *In vivo* tumor model

Female C57BL/6 mice aged 4–6 weeks were subcutaneously injected with 2 x 10^6^ Panc02 sh-NC and sh-ETV4 cells to establish tumor models. Tumor volumes (calculated as volume = length x (width^2)/2) and mouse weights were measured twice weekly. Experiments were terminated when tumor volumes reached 2000 mm^3^, and mice were euthanized to collect tumor weights. All animal experiments were approved by the Ethics Committee of Sun Yat-sen University Cancer Center (Approval No.: 23040k).

#### 2.2.7 RT-qPCR

Total RNA was extracted from cells and tissues using an RNA extraction kit (GOONIE, Cat # 400-100). cDNA was synthesized using a cDNA synthesis kit (Vazyse, Cat# R333-01) following the manufacturer’s protocols. qPCR was conducted using SYBR Color qPCR Master Mix (Vazyse, Cat# Q331-02). Reactions were performed in triplicate for three independent experiments. Primers for qPCR analysis are shown in **Supplementary Table S2**.

#### 2.2.8 Immunohistochemistry

IHC staining was performed on paraffin-embedded sections of Panc02 sh-NC and sh-ETV4 mouse tumors using specific antibodies against ETV4 (Abcam, ab189826, China) and FGL1 (Proteintech, Cat# 16000-1-AP, China).

### 2.3 Statistical analyses

Statistical analyses were performed using GraphPad Prism6 and RStudio version 4.3.1. Differences of p < 0.05 were considered statistically significant.

## 3 Results

### 3.1 Pan-cancer expression of ETV4 mRNA and survival analysis of ETV4 in 33 types of cancers

**Figure 1:**
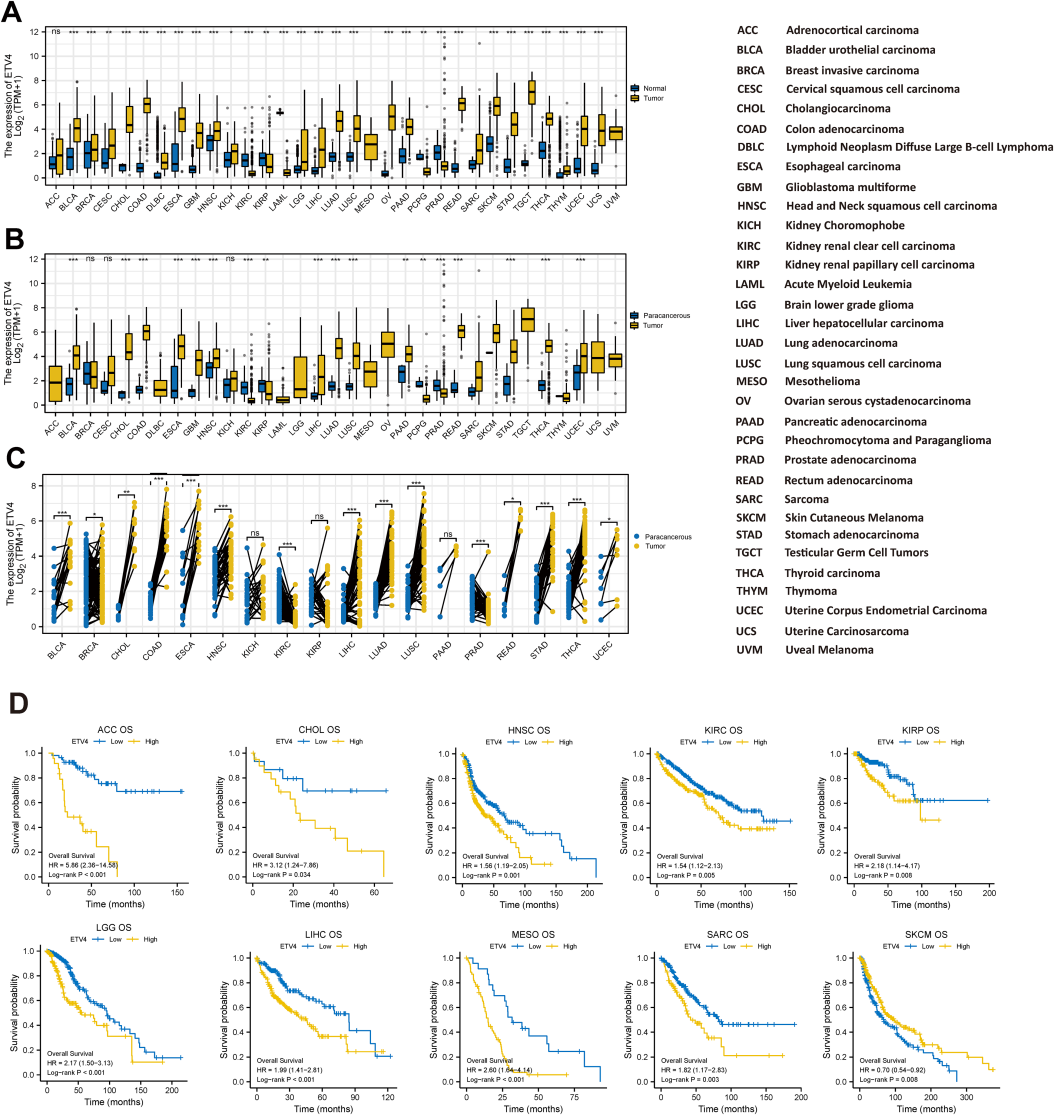
Expression of ETV4 mRNA and overall survival in Pan-cancer. **(A, B)** Unpaired sample analysis: **(A)** comparison of ETV4 expression between normal and tumor tissues, and **(B)** comparison between paracancerous and tumor tissues. **(C)** Paired sample analysis comparing ETV4 expression between tumor and adjacent paracancerous tissues. **(D)** Kaplan–Meier (KM) survival analysis of ETV4 expression in 10 specific cancer types. ns, no statistical significance. *p < 0.05, **p < 0.01, ***p < 0.001.

ETV4 was significantly elevated in the majority of tumors except ACC, PCPG, SARC, and PRAD. The expression of UVM and MESO could not be analyzed due to the lack of sufficient normal samples (**Figure 1A**). In comparison with paracancerous tissue, ETV4 mRNA expression was significantly higher in most digestive tract tumors (**Figure 1B**). In 18 types of cancers with paired sample analysis, ETV4 mRNA expression was generally increased, except in KIRC, PRAD, and KICH (**Figure 1C**).

The KM analysis was used to evaluate the prognostic value of ETV4. As illustrated in **Figure 1D** and **Supplementary Table S3**, the log-rank analysis indicated that ETV4 expression within 10 tumors as significantly associated with overall survival. Meanwhile, the high ETV4 group had greater OS than the ETV4 low expression group in SKCM. Interestingly, the low ETV4 groups showed better OS in ACC, CHOL, HNSC, KIRC, LIHC, KIRP, MESO SARC, and LGG (**Figure 1D**).

### 3.2 Genetic alteration of ETV4

**Figure 2:**
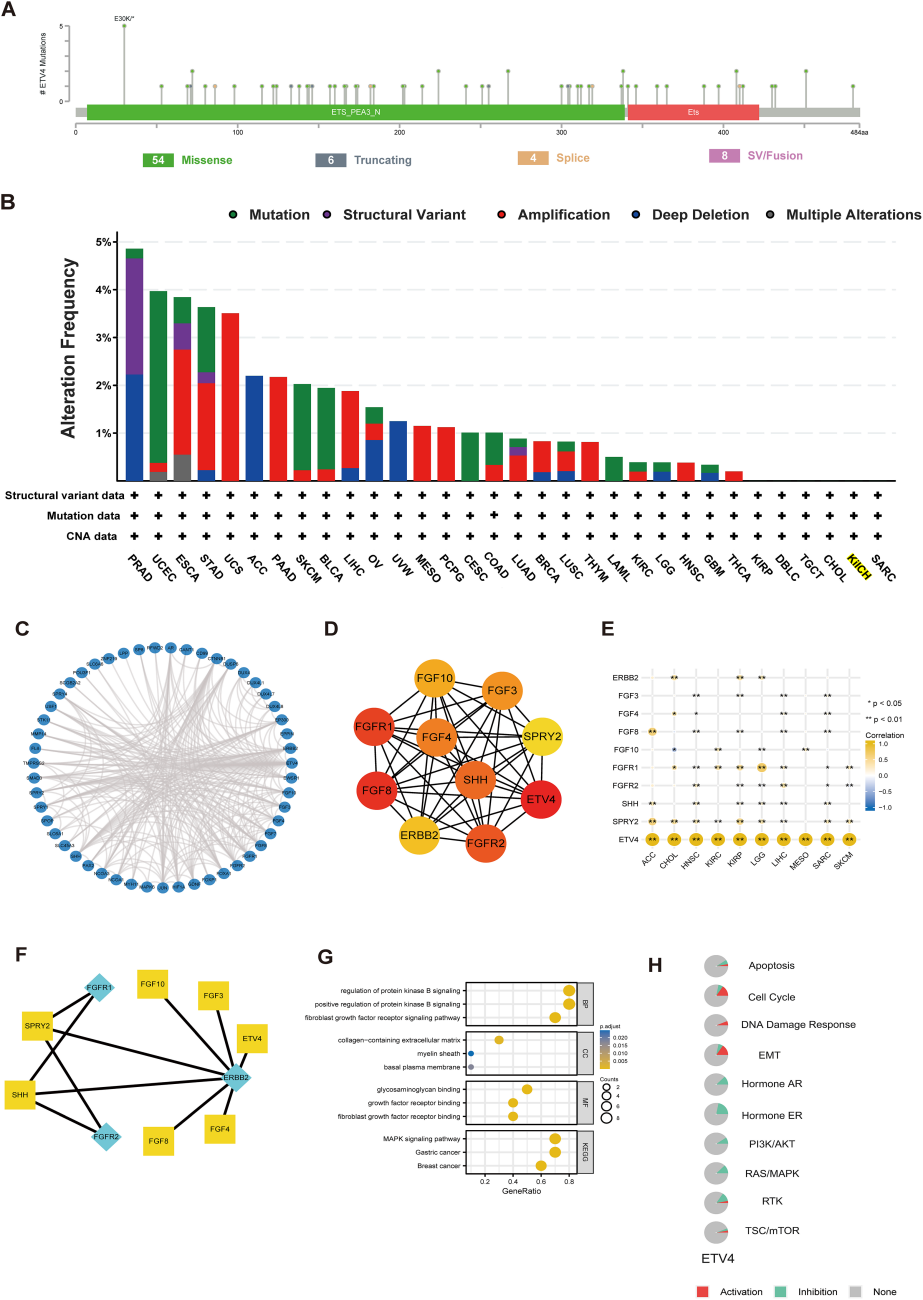
Genetic alterations, functional enrichment analysis, and PPI network of ETV4 in pan-cancer. **(A)** Mutation diagram showing the types and locations of ETV4 mutations. **(B)** Bar chart depicting the frequency of ETV4 mutations across different cancer types. **(C)** PPI network of ETV4. **(D, E)** Hub genes within the PPI network and their correlation with pan-cancer. **(F)** Signaling pathways reconstructed from the top ten hub genes, with yellow squares representing transcription factors and green diamonds representing receptors. **(G)** GO and KEGG pathway analysis associated with ETV4. **(H)** Correlation of ETV4 with pathway inhibition or activation. *p < 0.05, **p < 0.01.

We utilized the cBioPortal online tool to analyze genetic mutations in ETV4. A total of 72 mutation sites were identified between amino acids 0 and 484, with E30K emerging as the most frequently occurring mutation (**Figure 2A**). The predominant types of mutations included missense mutations, structural variants, and amplifications. Notably, genetic alterations in ETV4 were commonly observed in PRAD, UCEC, ESCA, STAD, and UCS (**Figure 2B**).

### 3.3 The functional enrichment and PPI of ETV4 in cancers

The STRING database was employed to identify genes closely associated with ETV4, facilitating the construction of PPI networks based on set thresholds (**Figure 2C**). The top 10 interacting genes are displayed in **Figure 2D**. Most of these genes, closely linked to ETV4 expression and prognosis, include receptors such as ERBB2, FGFR1, and FGFR2, with the remainder functioning as transcription factors (TFs) (**Figures 2E, F**). Through KEGG and GO analyses, ETV4 was found predominantly associated with growth factor-related pathways (**Figure 2G**). Pathways such as the Cell Cycle, DNA Damage Response, and Epithelial-Mesenchymal Transition (EMT) were activated by ETV4, while pathways like Hormone AR, Hormone ER, PI3K/AKT, RAS/MAPK, and RTK were found to be inhibited (**Figure 2H**).

### 3.4 Expression of ETV4 in various immune subtypes in pan-cancer

**Figure 3:**
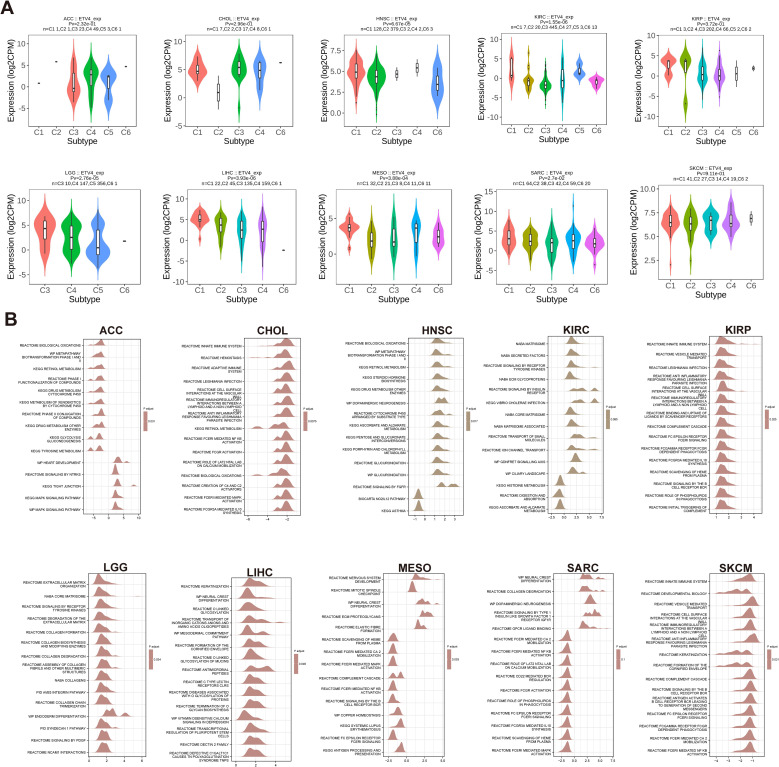
Correlation between ETV4 expression and immune subtypes, and Gene Set Enrichment Analysis (GSEA) pathways in 10 cancers. **(A)** Correlation between ETV4 expression and immune subtypes across 10 cancers. Immune subtypes include C1: wound healing, C2: IFN-γ dominant, C3: inflammatory, C4: lymphocyte depleted, C5: immunologically quiet, and C6: TGF-β dominant. **(B)** Top 15 GSEA pathways associated with ETV4 expression in 10 cancers. The X-axis represents logFC distributions of core molecules, while the Y-axis denotes the gene sets.

Building on previous findings that ETV4 is associated with overall survival (OS) in 10 tumor types, we extended our analysis to the expression of ETV4 across immune subtypes in these and an additional 23 tumor types. We observed variable ETV4 expression across different immune subtypes, particularly in HNSC, KIRC, LGG, LIHC, and MESO (**Figure 3A**). Notably, significant differential expression of ETV4 was evident in the immune subtypes of COAD, PRAD, STAD, and TGCT (**Supplementary Figure S1**).

### 3.5 Gene set enrichment

Analysis of gene set enrichment revealed common pathways influenced by ETV4, including the innate immune system, MAPK signaling pathway, metabolism, biological oxidations, and pathogen infection. These findings suggest that ETV4 plays a significant role in regulating tumor growth, metabolism, immune responses, and pathogen interactions across various cancers (**Figure 3B**).

### 3.6 Immunogenomic analyses of ETV4 in pan-cancer

**Figure 4:**
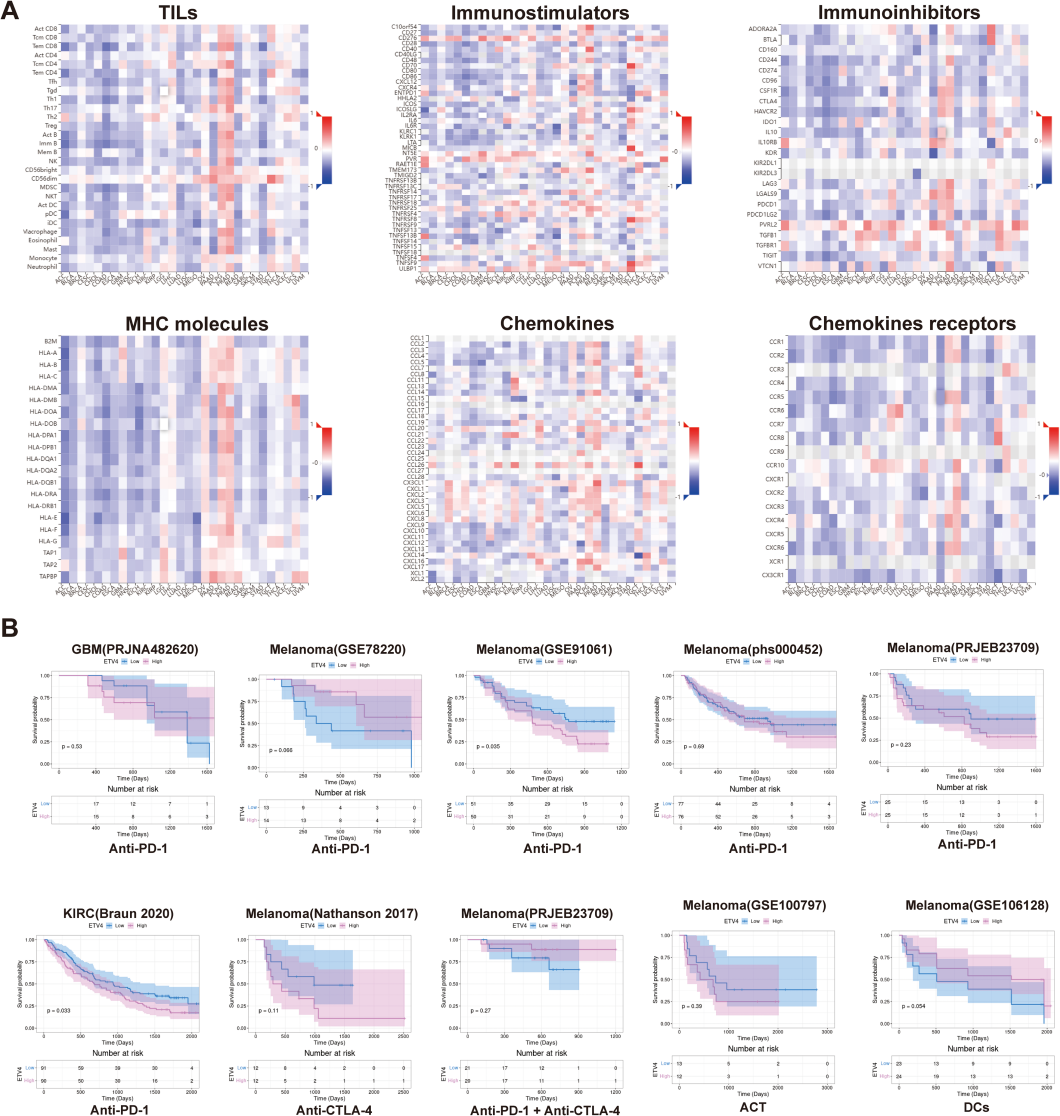
Correlation of ETV4 with immune-related genes and prediction of immunotherapy response in pan-cancer. **(A)** Correlation analysis between ETV4 and immune-related genes across various cancer types. **(B)** Prediction of immunotherapy response based on ETV4 expression in pan-cancer.

We utilized heat maps constructed with data on immune cells and immune factors to investigate the relationship between ETV4 expression and immune infiltration, as well as immune regulation across various cancers. In most tumors, ETV4 exhibited a negative association with the majority of tumor-infiltrating lymphocytes (TILs). However, in OV, PCPG, and PRAD, ETV4 was positively correlated with most TILs. For immunostimulatory molecules, a significant positive correlation with ETV4 was observed in LIHC, OV, PCPG, PRAD, and TGCT. Conversely, negative correlations were noted in BLCA, COAD, and STAD. Similarly, with respect to immunoinhibitors, ETV4 demonstrated positive relevance in PCPG and PRAD. Regarding MHC molecules, ETV4 showed a positive association with OV, PCPG, and PRAD, but was negatively associated with the majority of other tumors. The pattern persisted with chemokines, where ETV4 was positively correlated in OV, PCPG, and PRAD. In contrast, for chemokine receptors, ETV4 generally showed a negative correlation across most cancers (**Figure 4A**).

### 3.7 Prediction of immunotherapy response of ETV4

Utilizing datasets from the TIGER database, we assessed the potential of ETV4 to predict responses to immunotherapy. Our analysis indicates that ETV4 may serve as a valuable predictor of immunotherapy outcomes, particularly in melanoma and KIRC. Notably, higher levels of ETV4 expression were associated with a poor response to PD1 targeting therapies. However, ETV4 did not show predictive value for immunotherapy responses in other cancer types, which could be attributed to variations in treatment modalities and the limited availability of treatment data (**Figure 4B**).

### 3.8 Impact of ETV4 on tumor cell proliferation, migration, and apoptosis

**Figure 5:**
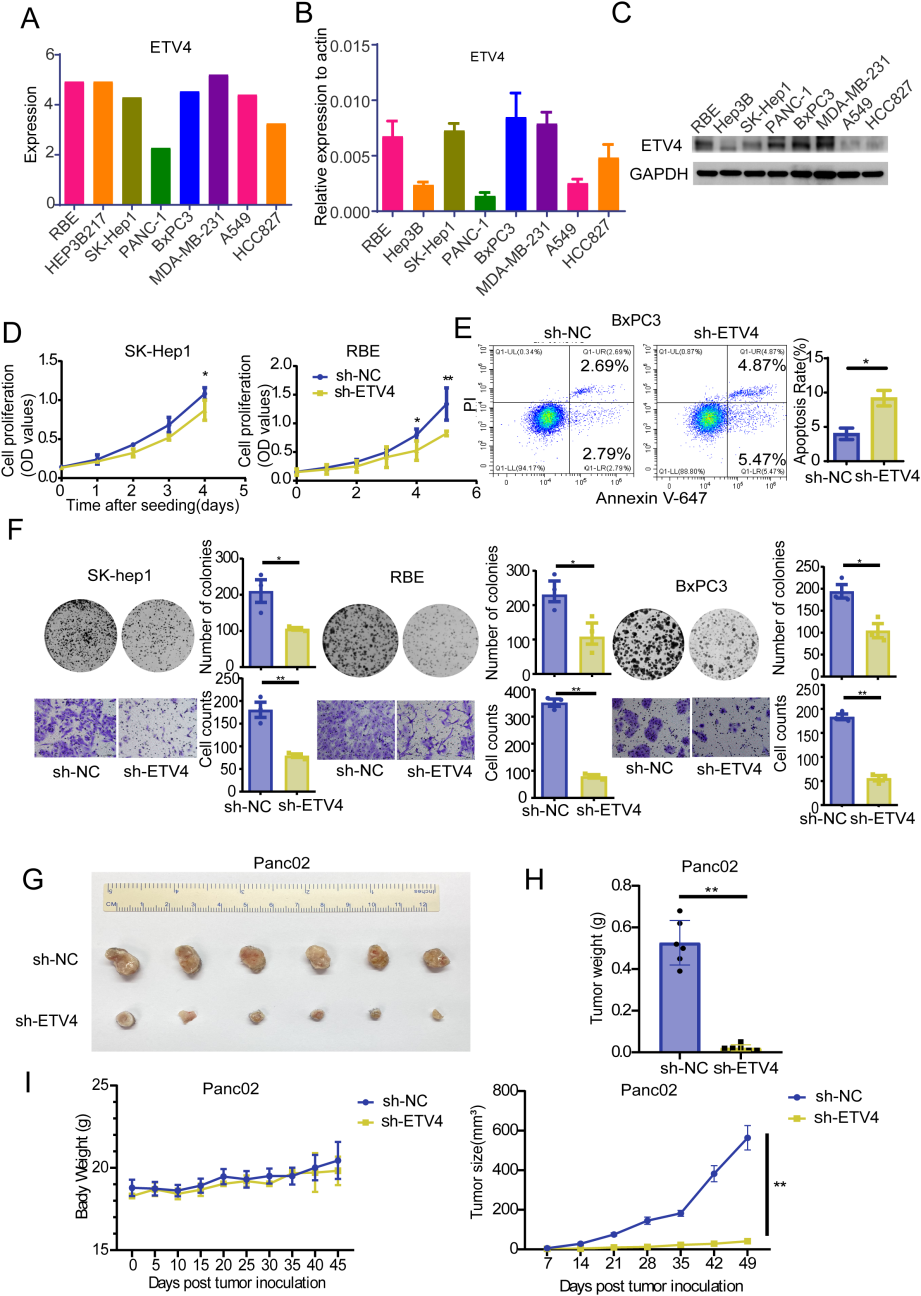
Phenotypic validation of ETV4 in digestive cancers. **(A)** ETV4 expression levels in cell lines from the CCLE database. **(B)** RT-qPCR validation of ETV4 expression in selected cell lines. **(C)** Western blot validation of ETV4 protein expression in selected cell lines. **(D)** CCK8 assay assessing the effect of ETV4 on proliferation in SK-Hep1 and RBE cells. **(E)** Flow cytometry analysis of the effect of ETV4 knockdown on apoptosis in BxPC3 cells. **(F)** Cell clonogenic and transwell assays evaluating the impact of ETV4 knockdown on proliferation and migration in SK-Hep1, RBE, and BxPC3 cells. **(G)** *In vivo* experiment showing representative images of tumors in C57BL/6 mice inoculated with 2×10^6 panc02 sh-NC or sh-ETV4 cells. **(H)** Statistical analysis of tumor weight in mice. **(I)** Graphical representation of tumor weight and tumor size changes in mice over time. *p < 0.05, **p < 0.01.

ETV4 mRNA and protein are broadly expressed across a wide range of human tissues and organs (**Supplementary Figure S2A**). According to data from the HPA and GTEx databases, ETV4 mRNA is predominantly expressed in the pituitary gland, esophagus, parathyroid gland, pancreas, breast, thyroid gland, gallbladder, skin, and duodenum (**Supplementary Figure S2B**). Protein expression data derived from the HPA database indicate that ETV4 protein is primarily enriched in the cerebral cortex, hippocampus, parathyroid gland, oral mucosa, duodenum, gallbladder, testis, placenta, and tonsil (**Supplementary Figure S2C**). To further validate the role of ETV4 in various cancers, we examined ETV4 expression across multiple tumor cell lines using the CCLE database (**Figure 5A**). RT-qPCR analysis confirmed high levels of ETV4 expression in BxPC3 (pancreatic cancer), MDA-MB-231 (breast cancer), RBE (intrahepatic cholangiocarcinoma), and SK-Hep1 (liver cancer) cells (**Figures 5B, C**). Representative IHC images from the HPA database further illustrate ETV4 protein expression in selected normal tissues and their corresponding tumor counterparts (**Supplementary Figure S2D**). ETV4 expression was specifically knocked down in BxPC3, RBE, and SK-Hep1 cells using shRNA, and successful knockdown was confirmed (**Supplementary Figure S3A**). CCK-8 and clonogenic assays demonstrated that ETV4 downregulation inhibited the proliferation of RBE and SK-Hep1 cells. Transwell assays revealed that ETV4 knockdown suppressed the migration of RBE, BxPC3, and SK-Hep1 cells. Furthermore, ETV4 knockdown increased apoptosis in BxPC3 cells (**Figures 5D, E**).

### 3.9 Effect of ETV4 on pancreatic cancer growth *in vivo*

To further explore the *in vivo* effects of ETV4, we established a stable Panc02 sh-ETV4 pancreatic tumor cell line in mice. Successful knockdown of ETV4 was confirmed at both the RNA and protein levels (**Supplementary Figure S3B**). The results indicated that ETV4 knockdown significantly slowed tumor growth without affecting mouse body weight (**Figures 5F–I**; **Figures 3C, D**).

### 3.10 Impact of ETV4 on immune-related molecules

**Figure 6:**
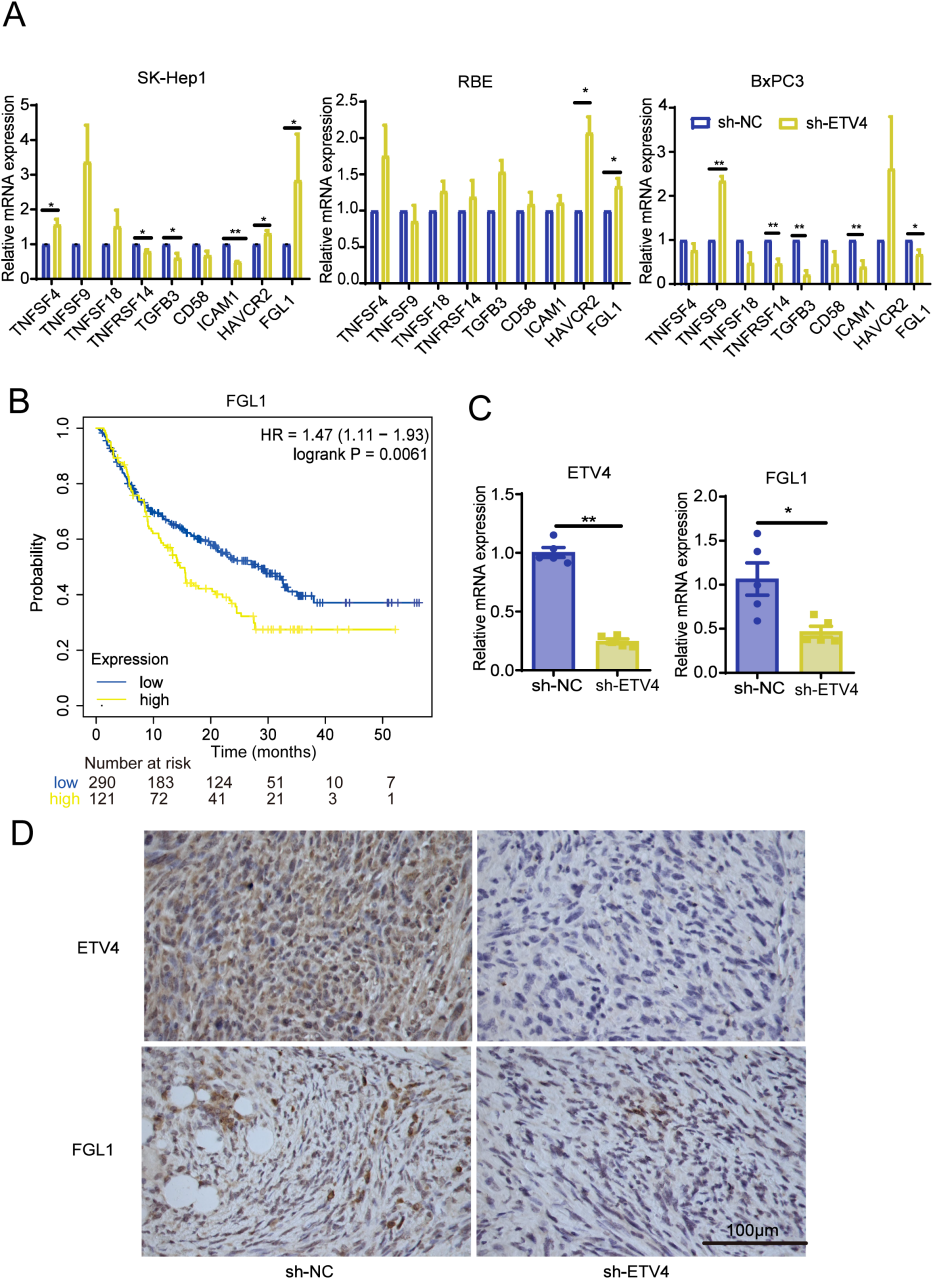
Validation of the effect of ETV4 on immune responses. **(A)** RT-qPCR analysis of changes in immune-related molecule expression in SK-Hep1, RBE, and BxPC3 cells following ETV4 knockdown. **(B)** Kaplan-Meier Plotter analysis showing the prognostic relationship between FGL1 expression and patient outcomes on anti-PD1 therapy. **(C, D)** RT-qPCR and immunohistochemistry were used to validate the expression levels of ETV4 and FGL1 in mouse tissues. *p < 0.05, **p < 0.01.

To assess the influence of ETV4 on immune-related molecules both *in vitro* and *in vivo*, we conducted RT-qPCR analyses. In SK-Hep1 cells, ETV4 downregulation led to increased expression of TNFSF4, HAVCR2, and FGL1, while TNFRSF14 and TGFβ3 expression decreased. In RBE cells, compared to the sh-NC group, ETV4 knockdown upregulated HAVCR2 and FCL1 expression. In BxPC3 cells, ETV4 knockdown increased TNFSF9 expression but decreased TNFRSF14, TGFβ3, ICAM1, and FGL1 expression (**Figure 6A**).

Kaplan-Meier Plotter analysis revealed that lower FGL1 expression correlated with longer OS in patients undergoing anti-PD1 therapy, including those with bladder cancer, glioblastoma, and melanoma (**Figure 6B**). Further RT-qPCR analysis of mouse tumor tissues demonstrated that knockdown of the ETV4 gene resulted in reduced FGL1 expression and no significant changes in HAVCR2, consistent with the results observed in BxPC3 pancreatic cancer cells (**Figure 6C**; **Supplementary Figure S3E**). Additionally, IHC analysis confirmed that ETV4 knockdown led to decreased FGL1 protein expression in mouse tissues (**Figure 6D**). To investigate the potential transcriptional regulatory relationship between ETV4 and FGL1, we conducted an in silico analysis using the NCBI and UCSC genome databases. By setting a minimum score threshold of 420, ETV4 was identified as a predicted transcription factor binding to the promoter region of FGL1, suggesting that ETV4 may directly regulate FGL1 transcription (**Supplementary Figure S4**).

## 4 Discussion

The death of tumor patients is more often attributed to metastasis than to the primary tumor, with metastasis being responsible for approximately 90% of cancer-related deaths (21). ETV4 has been implicated in the metastatic processes of many tumors (17, 22–25). Our research demonstrated that ETV4 is highly expressed in 29 different types of cancers, as well as in 18 pairs of matched tumor samples. Previous studies have confirmed its overexpression in various cancers (12, 26–29). Interestingly, we observed a decrease in ETV4 expression in prostate adenocarcinoma (PRAD). The study leaves the question of whether ETV4 expression is elevated in prostate cancer tissues uncertain, necessitating further research (30, 31). Although ETV4 overexpression leads to the development of mPIN, it does not directly result in cancer progression. ETV4 promotes prostate cell proliferation and metastasis through direct and p53-mediated downregulation of p21 (28). Kaplan-Meier analysis showed that high ETV4 expression correlates with poor prognosis across multiple cancers. Similarly, previous studies have linked ETV4 with shorter survival times in patients with breast cancer, pancreatic cancer, and colorectal cancer (12, 23, 27). For example, overexpression of ETV4 has been shown to activate the CXCL13/CXCR5 axis, promoting pancreatic cancer metastasis, and ETV4 expression serves as an independent prognostic factor influencing patient survival. This finding is significant for potential therapeutic strategies in the treatment of pancreatic cancer (23). In contrast, the high ETV4 group of SKCM showed better survival. This difference may be due to the unique biological and therapeutic properties of SKCM, which is known to have a high tumor mutational load (TMB) and high immunogenicity, and thus is particularly sensitive to immunotherapy and targeted therapies (32, 33). As prognostic data for TCGA primarily reflect survival after treatment, patients with high ETV4 may also have favorable genomic features, such as increased TMB or additional actionable mutations, that enhance treatment response and improve survival outcomes. Despite the favorable association with survival in transcriptomic datasets, functional studies suggest that ETV4 plays an oncogenic role in melanoma. For example, Zhang et al. identified ETV4 as a downstream effector of the melanoma-specific super-enhancer enh17, driving melanoma progression (34). Furthermore, Huang et al. demonstrated that pharmacological inhibition of pan-ETS factors, including ETV4, suppressed melanoma growth, further supporting its tumor-promoting potential (35). These findings underscore the context-dependent role of ETV4 across different tumor types. While ETV4 generally functions as a pro-tumorigenic factor and correlates with poor prognosis, exceptions such as SKCM highlight the need for integrated analyses that account for tumor-specific molecular features, treatment responses, and genomic landscapes.

We also observed that ETV4 had the highest frequency of gene alterations in PRAD, primarily structural variations and amplifications, consistent with previous studies. Jeremy et al. summarized the role of the ETS family in prostate cancer and found that chromosomal rearrangements leading to the overexpression of ETS gene family members are common in human prostate cancer (36). While the frequency of ETV1, ETV4, and ETV5 fusions is low, these fusion structures exhibit significant variability (37). ETS gene rearrangements represent early events that continue to be expressed in castrate-resistant and metastatic diseases. However, changes in ETS genes alone seem insufficient to induce cancer formation (38).

Enrichment analysis revealed that ETV4 is associated with various growth factor receptors and can influence the MAPK and PI3K/AKT pathways. The MAPK and PI3K/AKT/mTOR signaling pathways are crucial in regulating cell proliferation (39–41), differentiation (42), transformation (24), and apoptosis (43) by phosphorylating nuclear transcription factors and promoting cell cycle progression. These pathways are intimately connected to the development of inflammation, tumors, and other diseases, playing pivotal roles in tumor formation, erosion, and metastasis (44, 45). Yu et al. have determined that ETV4 can combine with the promoter of MMP1 to activate t its transcription, suggesting that the ETV4-related pathway may serve as a therapeutic target for colon cancer (46). ETV4, as part of the MAPK pathway, has also been shown to promote the occurrence and metastasis of esophageal squamous cell carcinoma (22). Additionally, ETV4 binds to the FOSL1 promoter, relying on PI3K-AKT signaling to directly upregulate FOSL1, leading to metastasis and disease progression in clear cell renal cell carcinoma (47). Moreover, the abnormal activation of the MAPK pathway can lead to the activation of oncogenic transcriptomes, where MAPK-dependent regulation of PEA3-ETS protein stability represents a critical signaling node for tumorigenesis and resistance to MAPK pathway inhibition (48). Decreased ectopic expression of ETS increases sensitivity to MEK and RAF inhibitors by affecting the reactivation of the RTK-RAS-MAPK pathway (49).

Our findings indicated that ETV4 enhances cancer cell migration and invasion by promoting epithelial-mesenchymal transition (EMT), which may trigger extracellular matrix degradation and anti-apoptotic pathways. ETV4 overexpression has been implicated in prostate carcinogenesis through the induction of EMT and cell proliferation (29). ETV4-driven EMT has also been described in lung cancer and thyroid carcinoma (50, 51). And similarly, its overexpression promotes breast cancer metastasis through Snail-induced EMT (17, 25).

Interestingly, our study found that knocking down ETV4 leads to an upregulation of FGL1 expression in RBE and SK-hep1 cells, while the opposite effect was observed in pancreatic cancer cells and tumors, where ETV4 knockdown resulted in the downregulation of FGL1 expression. This bidirectional regulation of FGL1 by ETV4 suggests a complex relationship between these two proteins, which may vary depending on the tumor type and cellular context. Specifically, the influence of ETV4 on FGL1 expression could be related to the baseline expression levels of FGL1 in different tumors. FGL1, a known ligand for the immune checkpoint receptor LAG3, plays a significant role in immune evasion by tumors (19, 52, 53). Elevated FGL1 levels have been associated with poor responses to PD1/PD-L1 blockade therapies, particularly in cancers like non-small cell lung cancer (NSCLC) and metastatic melanoma (54–56). In pancreatic cancer, the downregulation of FGL1 following ETV4 knockdown could potentially enhance the efficacy of immunotherapy by reducing immune suppression and improving T-cell activation. This suggests that targeting the ETV4-FGL1 axis may represent a promising strategy to overcome resistance to immune checkpoint inhibitors in pancreatic cancer.

In addition, we found that ETV4 has a predictive effect on the immunotherapy outcomes for melanoma and renal cell carcinoma, with high ETV4 expression indicating poorer responses to immunotherapy. ETV4 is closely related to immunity, as it activates genes involved in the recruitment of myeloid cells, leading to increased infiltration of bone marrow-derived suppressor cells and macrophages, while reducing the presence of NK cells and T cells (27). ETS proteins are also associated with immune infiltration in colorectal cancer (CRC), particularly involving M2 macrophages and cancer-associated fibroblasts (26). Interestingly, super-enhancers receive signals from the extracellular environment to induce PD-L1-mediated immune evasion by interacting with ETV4 (57). Our study further clarified that ETV4 has broader applicability across various tumors and confirmed that ETV4 expression is strongly linked to the biological processes of immune-related molecules and immune cells in most cancers. Moreover, our study revealed that ETV4 is closely related to MHC, immune activation, immunosuppression, chemokines, and chemokine receptors. Our results suggest that ETV4 is intricately involved in the immune invasion of tumor cells, impacts patient prognosis, and provides a novel target for drug development.

In conclusion, our study clarified the role of ETV4 in pan-cancer contexts from different perspectives, including its related signaling pathways, its relationship with immune cell infiltration, and its mutation sites. We demonstrated that ETV4 is involved in the activation or repression of key oncogenic pathways and is closely associated with immune cell infiltration and immunoregulatory processes. Functional assays further confirmed that ETV4 enhances tumor cell proliferation and migration while inhibiting apoptosis, reinforcing its role in tumor progression. Notably, we identified a potential transcriptional regulatory link between ETV4 and FGL1, an emerging immune checkpoint ligand. The proposed ETV4–FGL1 axis may contribute to tumor immune evasion and could affect patient responsiveness to immune checkpoint inhibitors. These findings provide a basis for exploring ETV4 as a potential therapeutic target to improve cancer immunotherapy.

However, several limitations must be acknowledged. First, a substantial portion of our analyses was based on public transcriptomic datasets and in silico predictions, which require validation in large, independent clinical cohorts. Second, although the association between ETV4 and FGL1 is supported by expression data and predicted promoter binding motifs, direct mechanistic evidence, such as chromatin immunoprecipitation and luciferase reporter assays, is still lacking. Third, the *in vivo* evidence supporting the immunomodulatory role of ETV4 and its regulation of FGL1 remains preliminary. Specifically, the extent to which the ETV4–FGL1 interaction contributes to immune evasion in immunocompetent models has not been fully elucidated and warrants further investigation. Finally, given the heterogeneity of ETV4 expression and function across cancer types, its utility as a universal biomarker or therapeutic target should be evaluated with caution.

Collectively, our findings provide new insights into the multifaceted role of ETV4 in tumor biology and immune regulation and lay the groundwork for future studies aimed at validating ETV4 as a biomarker and potential therapeutic target across cancers.

## Data Availability

The original contributions presented in the study are included in the article/**Supplementary Material**. Further inquiries can be directed to the corresponding authors.
