# Supplementary material for: Comprehensive analysis of the functional and immunological significance of ETV4 in pan-cancer and its validation in digestive tumors
Source: Front Immunol. 2025 May 21;16:1595850. doi: 10.3389/fimmu.2025.1595850 (PMC12133824; doi:10.3389/fimmu.2025.1595850)
Supplement: Supplementary file 1 [file DataSheet1.docx]

**Table S1 Sequences of oligonucleotides.**

| **Gene names** | **Sequences (5’to 3’)** |
| --- | --- |
| Human ETV4 -sh1 | GGATGAAAGCCGGATACTTGG |
| Human ETV4 -sh2 | GCTCGCTCCGATACTATTATG |
| Mouse ETV4-sh1 | GTCGGCCACAGAGGTGGATATT |
| Mouse ETV4 -sh2 | GGATGAAAGGCGGATACTTGG |
| Mouse ETV4-sh3 | GGTGATGGAGTGATGGGTTAT |

**Table S2 Sequences of primers.**

| **Region** | | **Forward primer sequence (5’to 3’)** | | **Reverse primer sequence (5’to 3’)** |
| --- | --- | --- | --- | --- |
| Human-ETV4 | | CAGTGCCTTTACTCCAGTGCC | CTCAGGAAATTCCGTTGCTCT | |
| Human-TNFRSF14 | | GTGCAGTCCAGGTTATCGTGT | | CACTTGCTTAGGCCATTGAGG |
| Human- TNFSF4 | | CCAGGCCAAGATTCGAGAGG | | CCGATGTGATACCTGAAGAGCA |
| Human- TNFSF9 | | GGCTGGAGTCTACTATGTCTTCT | | ACCTCGGTGAAGGGAGTCC |
| Human- TNFSF18 | | AGTGGCTCCCAATGCAAACTA | | TATACAGCCGCACCTCAAAAG |
| Human- HAVCR2 | | CTGCTGCTACTACTTACAAGGTC | | GCAGGGCAGATAGGCATTCT |
| Human- ICAM1 | | ATGCCCAGACATCTGTGTCC | | GGGGTCTCTATGCCCAACAA |
| Human- CD58 | | AGAGCATTACAACAGCCATCG | | ATCTGTGTCTTGAATGACCGC |
| Human-β-actin | AGAGCTACGAGCTGCCTGAC | | | AGCACTGTGTTGGCGTACAG |
| Human-GAPDH | TGACCACAGTCCATGCCATC | | | GACGGACACATTGGGGGTAG |
| Human-FGL1 | ATGGCAAAGGTGTTCAGTTTCA | | | ACAATCTGCATACTGCCTCTTG |
| Mouse-GAPDH | AGGTCGGTGTGAACGGATTTG | | | GGGGTCGTTGATGGCAACA |
| Mouse-FGL1 | CCCTGTCAGGAACTTTTCATCC | | | CGGTAGTAAACACCGTTCAGGT |
| Mouse-HAVCR2 | TCAGGTCTTACCCTCAACTGTG | | | GGCATTCTTACCAACCTCAAACA |
| Mouse-ETV4 | CGCACAGACTTCGCCTACG | | | CAGACATCATCTGGGAATGGTC |

**Table S3. Details of the prognostic K-M analysis of ETV4 in pan-cancer.**

|  | **Cancer** | **N** | **HR (95% CI)** | **P value** |
| --- | --- | --- | --- | --- |
|  | ACC | 79 | 1.63(1.33-1.99) | **<0.001** |
|  | BLCA | 413 | 1.04(0.94-1.15) | 0.419 |
|  | BRCA | 1082 | 1.04(0.93-1.16) | 0.485 |
|  | CESC | 306 | 1.11(0.97-1.27) | 0.147 |
|  | CHOL | 36 | 1.23(0.91-1.65) | 0.179 |
|  | COAD | 477 | 1.00(0.83-1.20) | 0.997 |
|  | DLBC | 48 | 0.80(0.44-1.45) | 0.470 |
|  | ESCA | 162 | 0.99(0.82-1.19) | 0.880 |
|  | GBM | 168 | 1.16(1.03-1.30) | **0.015** |
|  | HNSC | 501 | 1.14(1.02-1.28) | **0.026** |
|  | KICH | 64 | 1.33(0.73-2.41) | 0.355 |
|  | KIRC | 539 | 1.27(1.09-1.48) | **0.002** |
|  | KIRP | 288 | 1.16(0.96-1.38) | 0.131 |
|  | LAML | 140 | 1.20(0.82-1.75) | 0.344 |
|  | LGG | 527 | 1.16(1.07-1.26) | **<0.001** |
|  | LIHC | 373 | 1.18(1.08-1.28) | **<0.001** |
|  | LUAD | 526 | 0.97(0.88-1.08) | 0.612 |
|  | LUSC | 496 | 0.98(0.89-1.07) | 0.641 |
|  | MESO | 85 | 1.26(1.06-1.50) | **0.008** |
|  | OSCC | 328 | 1.07(0.92-1.23) | 0.384 |
|  | OV | 377 | 0.98(0.97-1.39) | 0.106 |
|  | PAAD | 178 | 1.16(1.04-2.38) | **0.032** |
|  | PCPG | 183 | 0.75(0.21-2.73) | 0.662 |
|  | PRAD | 499 | 1.16(0.85-1.58) | 0.339 |
|  | READ | 166 | 0.75(0.48-1.18) | 0.216 |
|  | SKCM | 456 | 0.93(0.84-1.04) | 0.217 |
|  | SARC | 263 | 1.20(1.08-1.33) | **<0.001** |
|  | STAD | 370 | 0.91(0.79-1.06) | 0.226 |
|  | TGCT | 139 | 1.40(0.53-3.70) | 0.496 |
|  | THCA | 510 | 0.73(0.54-0.99) | **0.049** |
|  | THYM | 118 | 0.89(0.48-1.66) | 0.717 |
|  | UCEC | 551 | 0.95(0.84-1.06) | 0.352 |
|  | UCS | 56 | 1.05(0.88-1.27) | 0.581 |
|  | UVM | 80 | 0.89(0.60-1.33) | 0.572 |


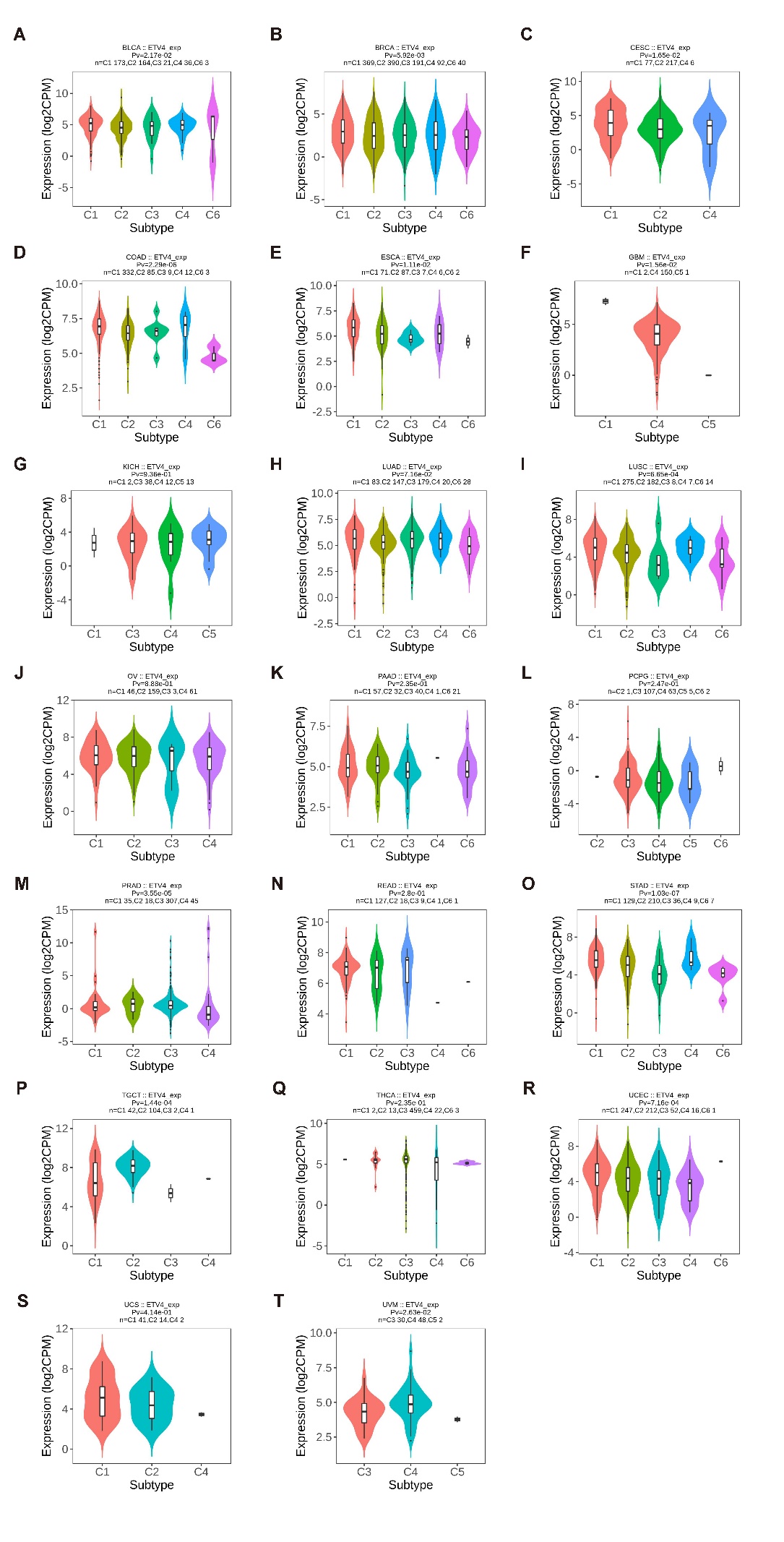


Supplementary Figure 1. Correlations between ETV4 expression and immune subtype in 20 cancers. (A) BLCA, (B) BRCA, (C) CESC, (D) COAD, (E) ESCA, (F) GBM, (G) KICH, (H) LUAD, (I) LUSC, (J) OV, (K) PAAD, (L) PCPG, (M) PRAD, (N) READ, (O) STAD, (P) TGCT, (Q) THCA, (R) UCEC, (S) UCS, (T) UVM.


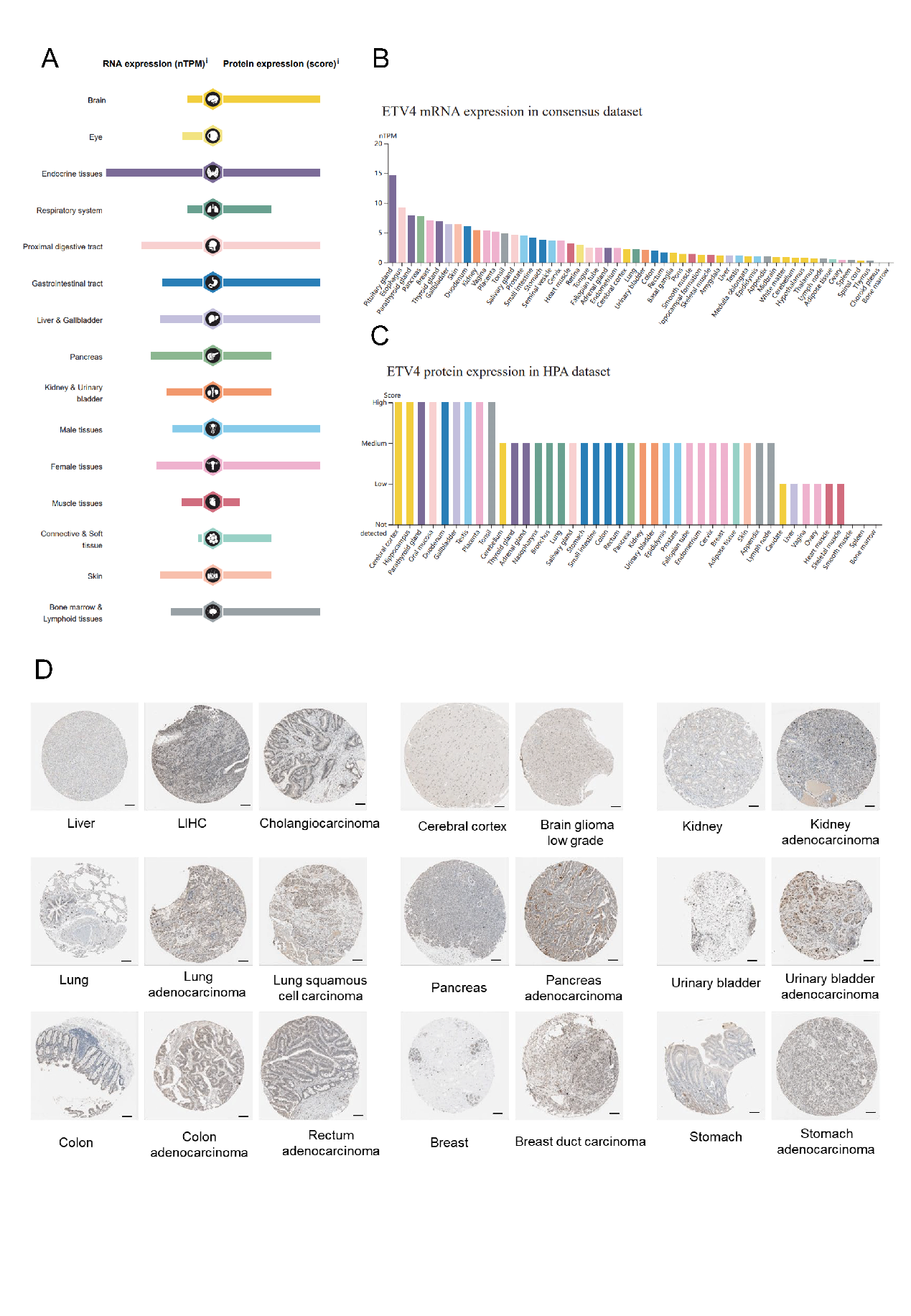


Supplementary Figure 2. ETV4 expression at the mRNA and protein levels in human organs and tissues. (A) Overview of ETV4 mRNA and protein expression across human tissues. (B) Summary of ETV4 mRNA expression in various human organs and tissues based on the Consensus dataset. (C) Summary of ETV4 protein expression in different human organs and tissues based on the Human Protein Atlas (HPA) dataset. (D) Representative immunohistochemistry images showing ETV4 protein expression in selected normal tissues and corresponding tumor tissues from the HPA database. Scale bar: 100 μm.


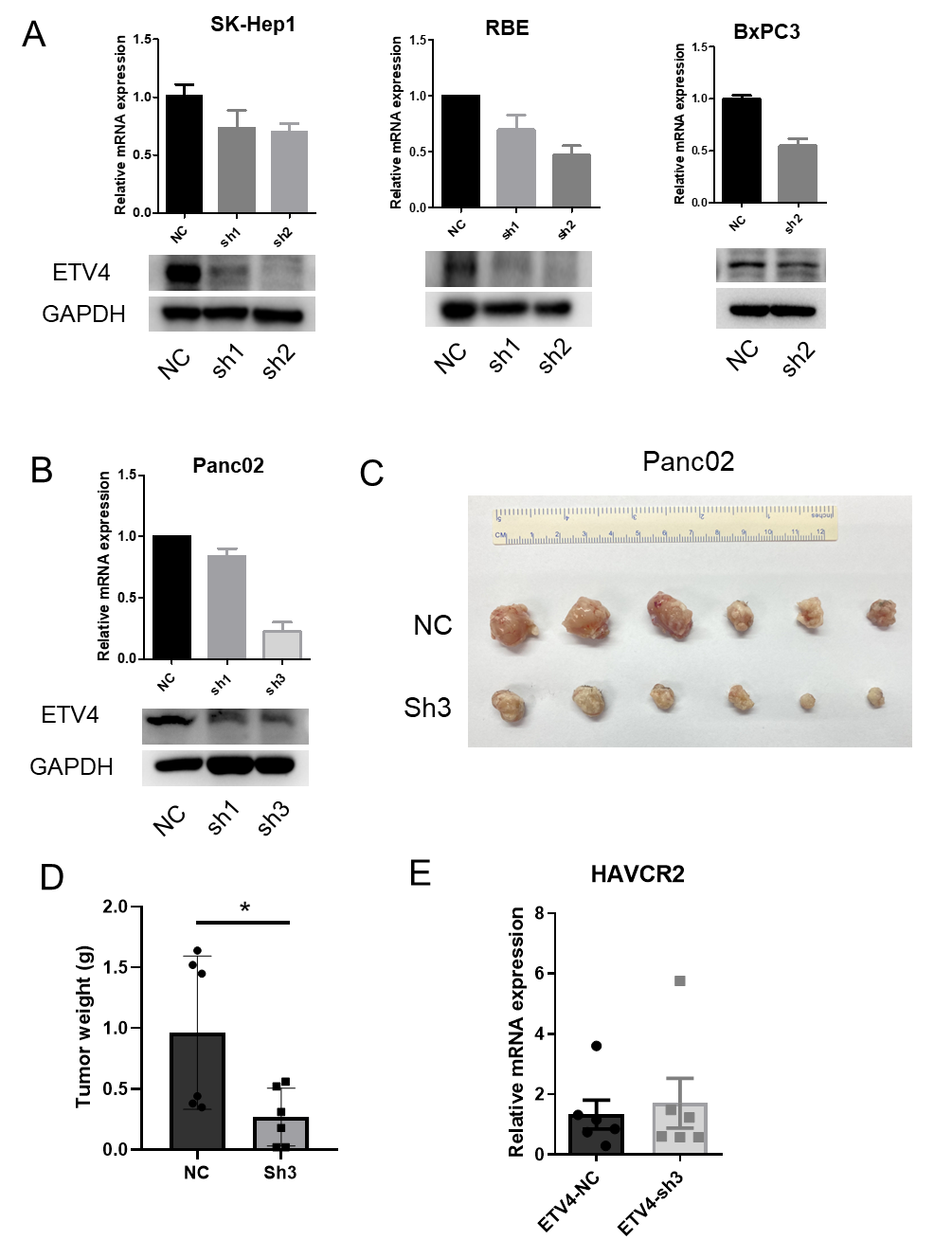


Supplementary Figure 3. Validation of ETV4 knockdown and its effects in vivo. (A-B) Successful knockdown of ETV4 was confirmed at both the RNA and protein levels. (C) In vivo experiment showing representative images of tumors in C57BL/6 mice inoculated with 2×10^6 panc02 sh-NC or sh-ETV4 cells (D) Statistical analysis of tumor weight in mice. (E) RT-qPCR analysis validating the expression levels of HAVCR2 in mouse tissues. *p < 0.05, **p < 0.01.


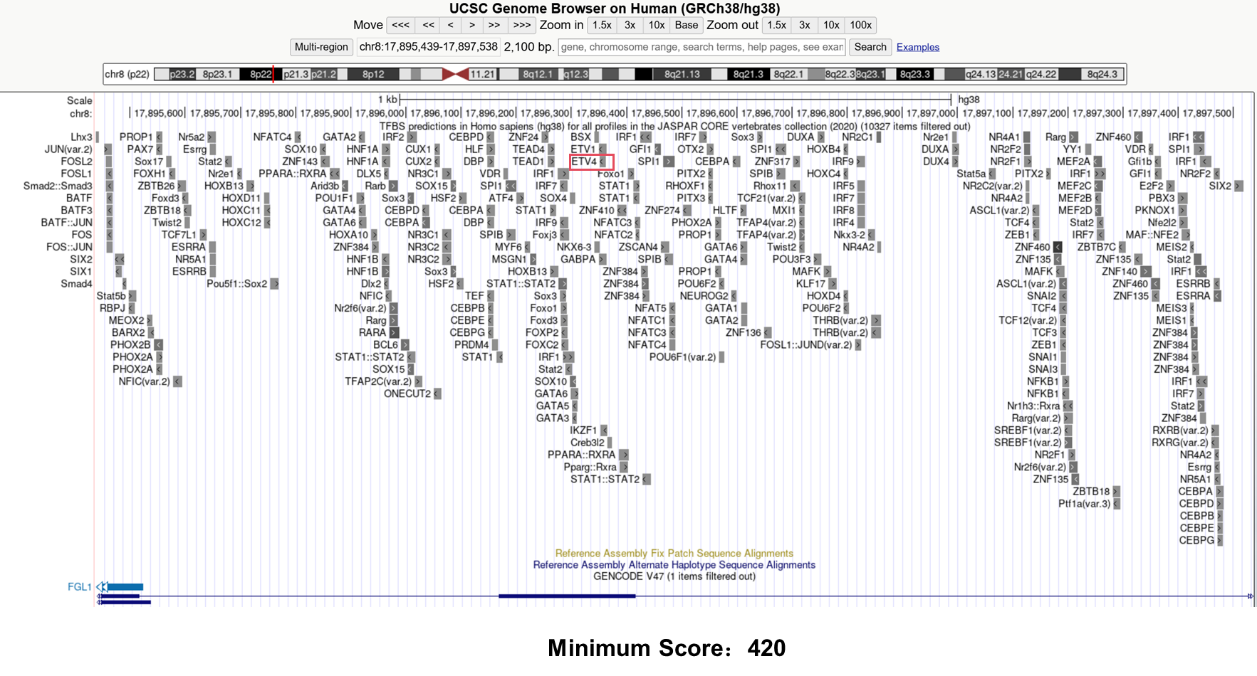


Supplementary Figure 4. Predicted transcriptional regulatory relationship between ETV4 and FGL1. Potential binding sites in the promoter region of FGL1 were identified using transcription factor prediction tools based on the NCBI and UCSC genome databases. The minimum score threshold was set to 420.
